# Treatment with Sildenafil and Donepezil Improves Angiogenesis in Experimentally Induced Critical Limb Ischemia

**DOI:** 10.1155/2017/9532381

**Published:** 2017-01-24

**Authors:** Ioana M. Constantinescu, Pompei Bolfa, Dan Constantinescu, Aurel I. Mironiuc, Claudia D. Gherman

**Affiliations:** ^1^Department of Surgery, Surgical Clinic II, “Iuliu Haţieganu” University of Medicine and Pharmacy Cluj-Napoca, 4-6 Clinicilor, 400006 Cluj-Napoca, Romania; ^2^University of Agricultural Sciences and Veterinary Medicine Cluj-Napoca, 3-5 Mănăştur, 400372 Cluj-Napoca, Romania; ^3^Department of Sugery, Surgical Clinic IV, “Iuliu Haţieganu” University of Medicine and Pharmacy Cluj-Napoca, 16-18-20 Republicii, 400015 Cluj-Napoca, Romania; ^4^Department of Medical Education, “Iuliu Haţieganu” University of Medicine and Pharmacy Cluj-Napoca, 23 Marinescu, 400337 Cluj-Napoca, Romania

## Abstract

*Objectives*. In this study, we aimed to demonstrate the role of sildenafil (an antagonist of phosphodiesterase type 5 (PDE-5)) and donepezil (a specific and reversible inhibitor of acetylcholinesterase (Ach)) in increasing ischemia-induced angiogenesis.* Method*. Critical limb ischemia was induced by ligation of the common femoral artery followed by ligation of the common iliac artery. The operated animals were divided into 3 groups: receiving sildenafil, receiving donepezil, and surgery alone; the contralateral lower limb was used as a negative control. The results were controlled based on clinical score and Doppler ultrasound. Gastrocnemius muscle samples were taken from all animals, both from the ischemic and nonischemic limb and were used for histopathological and immunohistochemical examination for the evaluation of the number of nuclei/field, endothelial cells (CD31), dividing cells (Ki-67), and vascular endothelial growth factor (VEGFR-3).* Results*. An increasing tendency of the number of nuclei/field with time was observed both in the case of sildenafil and donepezil treatment. The formation of new capillaries (the angiogenesis process) was more strongly influenced by donepezil treatment compared to sildenafil or no treatment. This treatment significantly influenced the capillary/fiber ratio, which was increased compared to untreated ligated animals. Sildenafil treatment led to a gradual increase in the number of dividing cells, which was significantly compared to the negative control group and compared to the ligation control group. The same effect (increase in the number of Ki-67 positive cells) was more obvious in the case of donepezil treatment.* Conclusion*. Donepezil treatment has a better effect in ligation-induced ischemia compared to sildenafil, promoting angiogenesis in the first place, and also arteriogenesis.

## 1. Introduction

Despite the progress made in the surgical, endovascular treatment of critical ischemia, as well as in the treatment of associated diseases and intervention on risk factors, this disease is associated with a high risk of amputation. In the absence of revascularization resources, stimulation of angiogenesis and arteriogenesis may have an important role.

Ischemia-induced arteriogenesis and subsequent angiogenesis are compensatory mechanisms that ensure tissue vascularization under these conditions [1].

The use of substances that release nitric oxide (NO) and activate endothelial NO synthase (eNOS), such as statins and VEGF-A, has proved to increase angiogenesis and arteriogenesis under critical ischemia conditions [2, 3]. However, substances that release nitric oxide can also have negative effects. NO excess associated with the lack of eNOS coupling results in the formation of superoxide and peroxynitrite radicals, which leads to the extension of the surface of affected tissues in ischemia-reperfusion syndrome [4].

Nitric oxide (NO) is known as a regulator of angiogenesis, participating in a number of cell reactions, including proliferation and survival of endothelial cells, increase of their mobility, and activation of mechanisms required for angiogenesis [1, 3, 5].

Sildenafil is an antagonist of phosphodiesterase type 5 (PDE-5), which inhibits cGMP catabolism, stimulating the activation of mechanisms that involve cGMP. Thus, sildenafil is used in the treatment of erectile dysfunction, pulmonary hypertension, congestive heart disease, and diabetic neuropathy [6–8]. Furthermore, sildenafil had promising results in the treatment of ischemia-reperfusion lesions at cardiac level and proved to be useful in the treatment of experimental embolic stroke in rats [4, 9, 10].

Donepezil is a specific and reversible inhibitor of acetylcholinesterase (Ach), which increases the bioavailability of acetylcholine, a neurotransmitter present in the peripheral central nervous system. This is used in the treatment of Alzheimer's disease, being known as a drug that improves cognitive function [11]. The involvement of Ach in the prevention of myocardial cell apoptosis has brought into discussion its effect as a trophic factor [12].

In this study, we aimed to demonstrate the role of sildenafil and donepezil in increasing ischemia-induced angiogenesis and we tried to compare the angiogenic effect of the two preparations that have a common point in their mechanism of action. While sildenafil is a phosphodiesterase inhibitor, with direct implication in NO action on the vascular system, donepezil, as an AChE inhibitor, has an indirect role in NO synthesis, using the cholinergic mechanism. Also, NO is supposed to be one of the signaling molecules induced by Ach [13]. NO has actions on revascularization of the ischemic limb: increased NO levels were found in the ischemic limb, the lack of NO impairs the process of revascularization, and the improvement of NO levels improves revascularization [14].

## 2. Material and Methods

In our experimental model, we used adult Wistar rats, with a weight of 180–200 grams. The animals received a standard diet, except for the immediate preoperative period. The animals were kept under standard laboratory conditions, according to protocols in use, at the Experimental Research Biobase of “Iuliu Hațieganu” University of Medicine and Pharmacy Cluj-Napoca. All experimental designs and surgical procedures were approved by the Ethical Commitee of “Iuliu Hațieganu” University of Medicine and Pharmacy Cluj-Napoca (Permit number 681/2012).

### 2.1. Experimental Model

Critical limb ischemia was induced by ligation of the common femoral artery, proximal to the origin of the deep femoral artery (day 0), followed by ligation of the common iliac artery (day 4), according to a previously published model (Lejay, 2012). The rats were anesthetized with ketamine 100 mg/kg and xylazine 8 mg/kg.

*Control of ischemia* was performed on day 5 based on clinical score (0 = normal mobility, 1 = pallor, limping, 2 = gangrenous tissue limited to less than half of the leg, without lower limb necrosis, 3 = gangrenous tissue limited to less than half of the leg, with lower limb necrosis, 4 = gangrenous tissue extended to more than half of the leg, and 5 = extensive lower limb necrosis) [1] and imaging score, by Doppler ultrasound.

### 2.2. Administration of Medication

The animals were divided into 3 groups:

1. operated rats receiving sildenafil, subdivided into 4 groups depending on the time of sacrifice: at 7 days, at 14 days, at 21 days, or at 30 days after ligation;
2. operated rats receiving donepezil, subdivided into 4 groups depending on the time of sacrifice: at 7 days, at 14 days, at 21 days, or at 30 days after ligation;
3. rats undergoing surgery alone (ligation control), sacrificed at 7 days, at 14 days, at 21 days, or at 30 days after ligation.

Starting with day 5, animals in group I were treated postoperatively with a therapeutic dose of sildenafil, 10 mg/kg body weight/day, by oropharyngeal gavage, for 30 days (day 5–day 34); we utilized sildenafil powder resulting from 100 mg pills dissolved in drinking water, to a final concentration of 40 *μ*g/mL. A resulting dose of 10 mg/kg/day of sildenafil was achieved, to reach a therapeutic level that would be comparable to that in humans, because of the short half life in rats (1 hour in rats versus 4 hours in humans) [15].

In group II, the operated animals were treated postoperatively with a therapeutic dose of donepezil, 5 mg/kg/day, by oropharyngeal gavage, for 30 days (day 5–day 34); we utilized donepezil powder resulted from 10 mg pills dissolved in drinking water, to a final concentration of 50 *μ*g/mL. A resulting dose of 5 mg/kg/day of donepezil was achieved, to reach a therapeutic dosing level that would be comparable to that in humans. This dose was initially determined to clearly show the expected effects without producing adverse effects in rats [16].

The treatment was administered by gavage for 30 days.

The (nonischemic) contralateral lower limb was used as a negative control.

*The results of treatment* were controlled on days 7, 14, 21, and 30, based on clinical score and Doppler ultrasound, with a VisualSonics® Vevo® 2100 machine.

At the end of each experimental period (7, 14, 21, and 30 days), the rats were euthanized and gastrocnemius muscle samples were taken from all animals, both from the ischemic and the nonischemic limb. The muscles were dissected and introduced for fixation in 10% neutral buffered formol saline for 24 hours. For histopathological examination, the muscle tissue samples were cross-sectioned into slices about 4 mm thick and were subsequently processed by paraffin embedding.

After 4 micrometer* serial sections* were cut, some of these sections were used for histopathological examination by hematoxylin-eosin (H&E) staining, and some were sectioned on slides pretreated with adhesive for immunohistochemical examination. For the negative control group, gastrocnemius muscle sections from the nonligated limb of each rat in each ligation group were used.

The protocol followed for* H&E (hematoxylin and eosin) staining* consisted of deparaffinization of the samples, by their immersion in two xylene baths for 3 minutes, hydration of the samples in 3 successive ethyl alcohol baths (100%: 3 minutes, 90%: 3 minutes, and 70%: 3 minutes), followed by immersion in distilled water, then, immersion in Gill 2 hematoxylin for 2 minutes, washing in a tap water bath for 3 minutes which will produce color change, immersion in eosin-phloxine solution for 1 minute, and tap water washing. Subsequently, the process continues with dehydration of the samples in 3 successive ethyl alcohol baths (70%: 3 minutes, 90%: 3 minutes, and 100%: 3 minutes), clarification by immersion in xylene for 3 minutes, twice, and mounting (mounting medium), followed by microscopic examination.

*Immunohistochemical examination* (IHC) of paraffin samples: after the stage of deparaffinization and hydration (in two successive xylene baths, followed by absolute alcohol, 95% ethyl alcohol, 70% ethyl alcohol, and distilled water baths), the stage of antigen unmasking was conducted: with 10 mmol/L Tris buffer, 1 mmol/L EDTA, pH 9.0, for 20 minutes in hot bath (for CD31), with citrate buffer, pH 6.1, for 20 minutes (for Ki-67), and in hot bath with citrate buffer 0.01 mL/L, pH 6.0, for 60 minutes (for VEGFR-3), respectively. Then, the samples were incubated with the following primary antibodies: mouse anti-human CD31 monoclonal antibody (clone JC70A, Dako), diluted 1 : 30, for 30 minutes, at room temperature; mouse anti-human Ki-67 monoclonal antibody (clone MIB-1, Dako), diluted 1 : 100, for 20 minutes, at room temperature; mouse anti-human VEGFR-3 monoclonal antibody (clone KLT9), diluted 1 : 75, for 60 minutes, at room temperature. For each antibody, a negative control was used, which consisted of tissue samples to which only the diluent without the antibody was added. After washing with PBS, a secondary detection kit, Novostain Universal Detection Kit (Novocastra), was used. Thus, the sections were incubated with a universal biotinylated secondary antibody (10 minutes), washed in PBS (5 minutes), incubated with the streptavidin/peroxidase complex (10 minutes), washed in PBS (5 minutes), followed by incubation with diaminobenzidine (DAB) until the appearance of a slightly brownish color of the preparations, washed in tap water (5 minutes), counterstained with Gill 2 hematoxylin, dehydrated (in successive 70%, 95%, and 100 alcohol, then xylene baths), and mounted with anhydrous mounting medium (Neo-Mount®).

The histopathological and immunohistochemical preparations were examined and photographed using an Olympus calibrated image acquisition and processing system, an Olympus BX51 microscope equipped with a DP 25 digital camera and the Olympus Cell B image acquisition and processing program. Multiple quantifications were performed and their mean was calculated to obtain one value for each animal and each group, respectively.

### 2.3. Quantification of the Number of Nuclei per Microscopic Field

In the H&E stained preparations, the number of nuclei in structures compatible with capillaries in 9 different fields (magnification ×400) was evaluated, according to a previously published technique [16] (Kakinuma et al., 2010).

### 2.4. Capillary Density Analysis

Capillary density (number of capillaries per number of muscle fibers) was determined in 10 different fields (magnification ×400), from each section from each animal, and was expressed as the mean number of CD31 positive cells per mm^2^. To avoid underestimation or overestimation of muscle density due to muscle atrophy or interstitial edema, the capillary/muscle fiber ratio was also expressed [17] (Khazaei and Salehi, 2012).

### 2.5. Assessment of Endothelial Cell Proliferation

Endothelial cell proliferation was immunohistochemically assessed using a marker for cell proliferation (Ki-67) and a marker for endothelial cell growth factor receptor 3 (VEGFR-3). Ki-67 and VEGFR-3 positive vessels were quantified by an adapted method used by Brieger et al. (2004) [18], similarly to CD31 quantification (10 different fields with a magnification ×400 from each section from each animal, expressed as the mean number of positive cells per 1 mm^2^).

**Figure 1:**
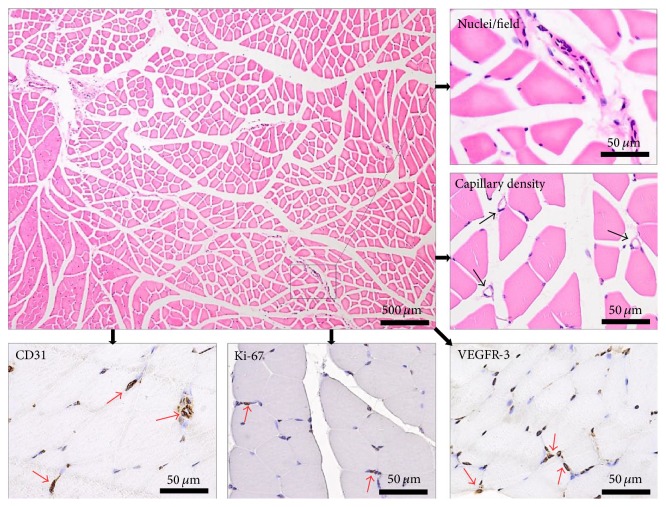
Techniques used for the histopathological study. Gastrocnemius muscle cross-sections: H&E staining for the evaluation of the number of nuclei/field; immunohistochemical staining for endothelial cells (CD31), dividing cells (Ki-67) and vascular endothelial growth factor (VEGFR-3).

Figure 1 illustrates the techniques used for the histopathological study.

### 2.6. Statistical Analysis

All quantification values were expressed as mean ± standard deviation. The normal (or parametric) distribution of data used for statistical analysis was verified using the Shapiro-Wilk normality test. The results were analyzed with the one-way ANOVA test, followed by the *t*-test with the Bonferronni correction. *P* values < 0.0125 were considered statistically significant for the *t*-test after the Bonferroni correction. The 4 experimental groups were compared at each of the four experimental times (7, 14, 21, and 30 days). Analysis was conducted with the Microsoft Excel 2010 statistical analysis program.

## 3. Results

### 3.1. Quantification of the Number of Nuclei per Microscopic Field

**Figure 2:**
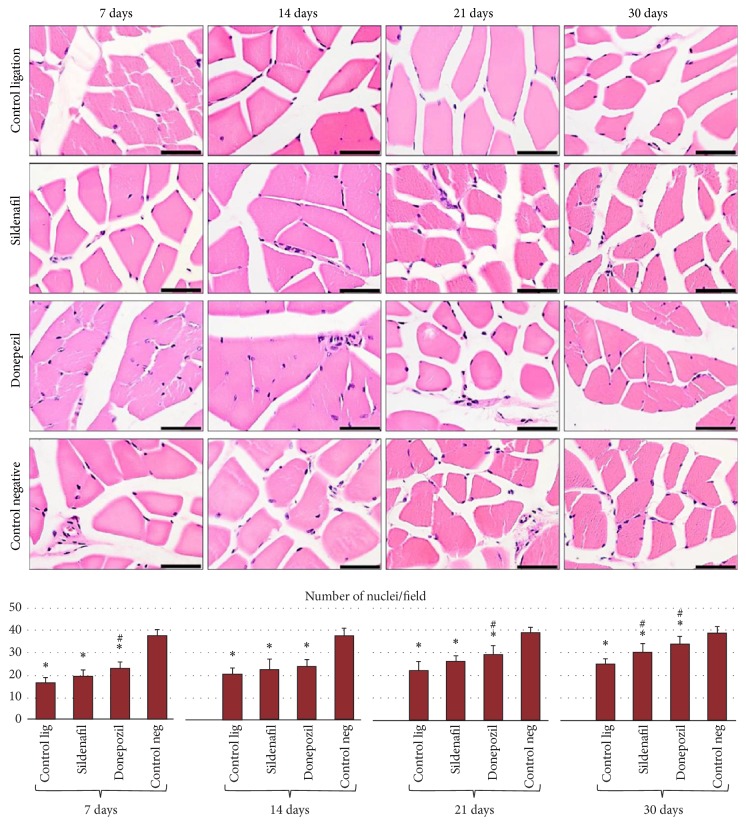
Evaluation of the number of nuclei/field in gastrocnemius muscle sections from Wistar rats, at the 4 experimental times (H&E staining, scale bar is 50 *μ*m). Quantification of the number of nuclei/field: the graph shows the mean and standard deviation for each experimental variant. Statistical differences are marked: ^*∗*^*P* < 0.0125 compared to the negative control group, ^#^*P* < 0.0125 compared to the ligation control group.

The number of nuclei in structures compatible with capillaries per HPF (High Power Field) in the H&E stained sections was calculated (Figure 2). There were statistically significant differences between the 4 experimental groups, at all four time points of sacrifice of the rats (Anova, *P* < 0.001).

It could be seen that the smallest number of nuclei in structures compatible with blood vessels per field was present in animals of the ligation control group, at all four experimental times compared to that of the negative control group (*P* < 0.0125).

Sildenafil treatment led to an increase of quantified nuclei/microscopic field, but this increase was statistically significant compared to rats of the ligation control group only at 30 days (*P* < 0.0125). At all four experimental times, the number of nuclei/field in the gastrocnemius muscle was significantly lower compared to animals of the negative control group. An increasing tendency of the number of nuclei/field with the increase of the time period elapsed from ligation was observed both in the case of sildenafil treatment and donepezil treatment, the highest values being recorded at 30 days.

Following donepezil treatment, the number of capillary structures was significantly lower compared to the negative control group at all four experimental times, but it was significantly higher compared to the ligation control group (without treatment) at 7, 21, and 30 days (*P* < 0.0125).

No statistically significant differences were found between sildenafil and donepezil treatment, although at all four experimental times the number of nuclei/field was higher after donepezil treatment.

### 3.2. Capillary Density Analysis

Neovascularization was assessed as capillary density (CD31 positive cells) per mm^2^ and as the capillary/muscle fiber ratio. There were statistical differences at all four experimental times between the 4 groups (Anova, *P* < 0.001).

**Figure 3:**
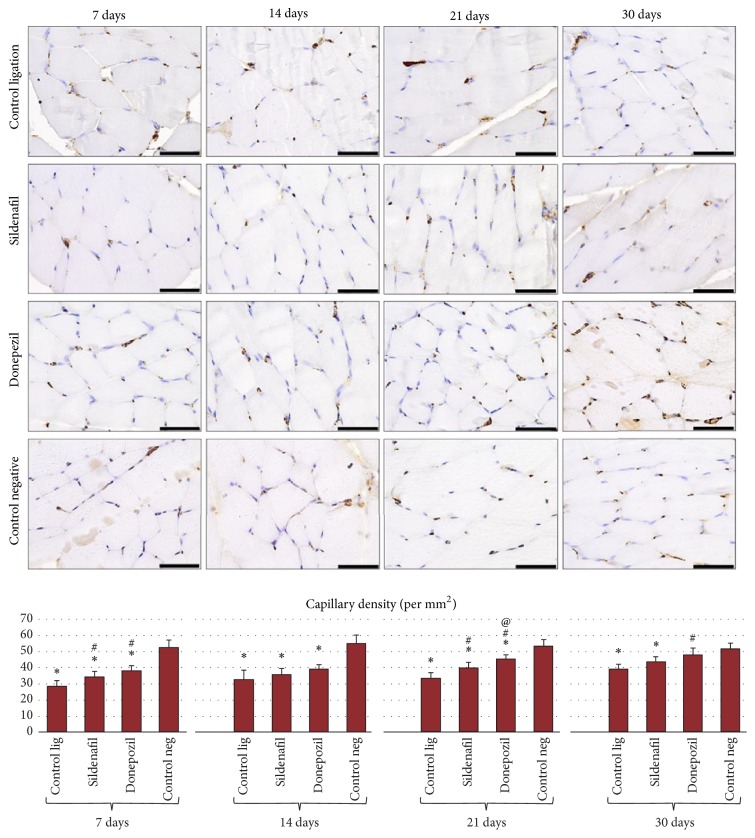
Histochemical evaluation of capillary density in gastrocnemius muscle sections from Wistar rats, at the 4 experimental times (scale bar is 50 *μ*m). Quantification of the number of CD31 positive cells/mm^2^: the graph illustrates the mean and standard deviation for each experimental variant. Statistical differences are marked: ^*∗*^*P* < 0.0125 compared to the negative control group, ^#^*P* < 0.0125 compared to the ligation control group, and ^@^*P* < 0.0125 compared to the sildenafil treated group.

In all three ligation groups (with or without treatment), an increase in the number of CD31 positive cells, proportional to the time elapsed, was found (Figure 3).

At all four experimental times, in the case of the ligation control group, capillary density/mm^2^ was significantly lower (*P* < 0.0125) compared to the negative control group (nonligated gastrocnemius muscle). Similarly, in the case of groups treated with sildenafil, capillary density was significantly lower (*P* < 0.0125) compared to the negative control group, but higher compared to the ligation control group. This increase was significant only at 7 and 21 days (*P* < 0.0125).

It was observed that the formation of new capillaries (the angiogenesis process) was more strongly influenced by donepezil treatment compared to sildenafil or no treatment. Thus, donepezil treatment led to a significant increase (*P* < 0.0125) in the number of capillaries/mm^2^ compared to the ligation control group at 7, 21, and 30 days and compared to sildenafil treatment at 21 days.

The analysis of the capillary/muscle fiber ratio showed differences between the 4 experimental groups at all four experimental times (Anova *P* < 0.001).

The same tendency as that found by capillary density analysis was evidenced: among the treatment groups, the capillary/muscle fiber ratio was the highest in the case of donepezil treatment. At 30 days, this treatment significantly influenced (*P* < 0.0125) the capillary/fiber ratio, which was increased at this time point compared to untreated ligated animals (ligation control group).

**Figure 4:**
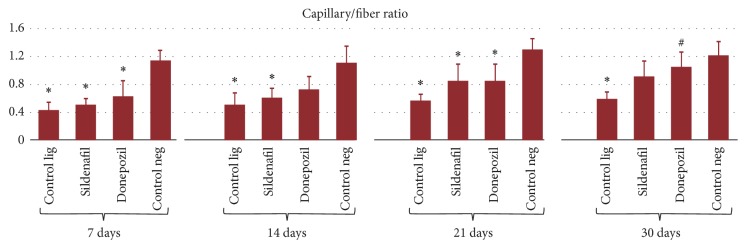
Evaluation of the capillary/muscle fiber ratio: the graph illustrates the mean and standard deviation for each experimental variant. Statistical differences are marked: ^*∗*^*P* < 0.0125 compared to the negative control group, ^#^*P* < 0.0125 compared to the ligation control group.

It was also found that the capillary/muscle fiber ratio gradually increased with the time elapsed from ligation, in all 3 ligation groups (Figure 4).

In animals of the ligation control group, without treatment, the capillary/muscle fiber ratio was significantly lower at all four experimental times (*P* < 0.0125) compared to the negative control group.

### 3.3. Assessment of Endothelial Cell Proliferation

Following immunohistochemical study, it was found that, in the gastrocnemius muscle sections from the nonligated limbs, the number of dividing (Ki-67 positive) cells was very low, unlike in the muscle sections from the ligated limbs. Significant differences were found at all four experimental times (Anova *P* < 0.001).

Sildenafil treatment led to a gradual increase in the number of dividing cells (at 7, 14, 21, and 30 days), which was significant compared to the negative control group (*P* < 0.0125) at 14, 21, and 30 days and compared to the ligation control group at 14 and 30 days.

**Figure 5:**
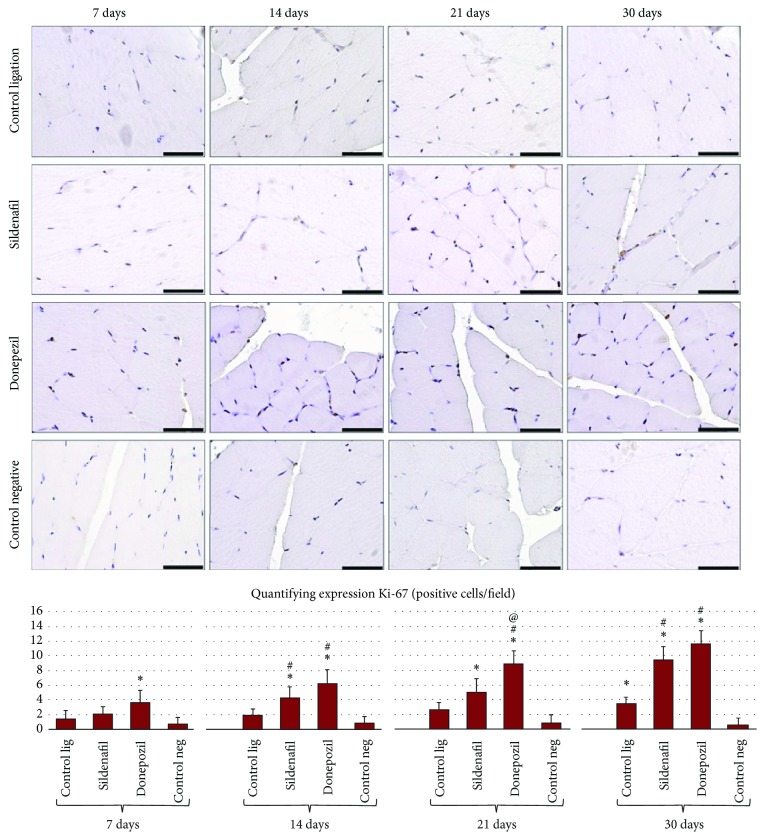
Histochemical evaluation of dividing cells in gastrocnemius muscle sections from Wistar rats, at the 4 experimental times (scale bar is 50 *μ*m). Quantification of the number of Ki-67 positive cells/field: the graph illustrates the mean and standard deviation for each experimental variant. Statistical differences are marked: ^*∗*^*P* < 0.0125 compared to the negative control group, ^#^*P* < 0.0125 compared to the ligation control group, and ^@^*P* < 0.0125 compared to the sildenafil treated group.

The same effect (increase in the number of Ki-67 positive cells) was more obvious in the case of donepezil treatment. Thus, the number of Ki-67 positive cells was significantly higher (*P* < 0.0125) at 7, 14, 21, and 30 days compared to the negative control group, at 14, 21, and 30 days compared to the ligation control group and at 21 and 30 days compared to the sildenafil treated group (Figure 5).

**Figure 6:**
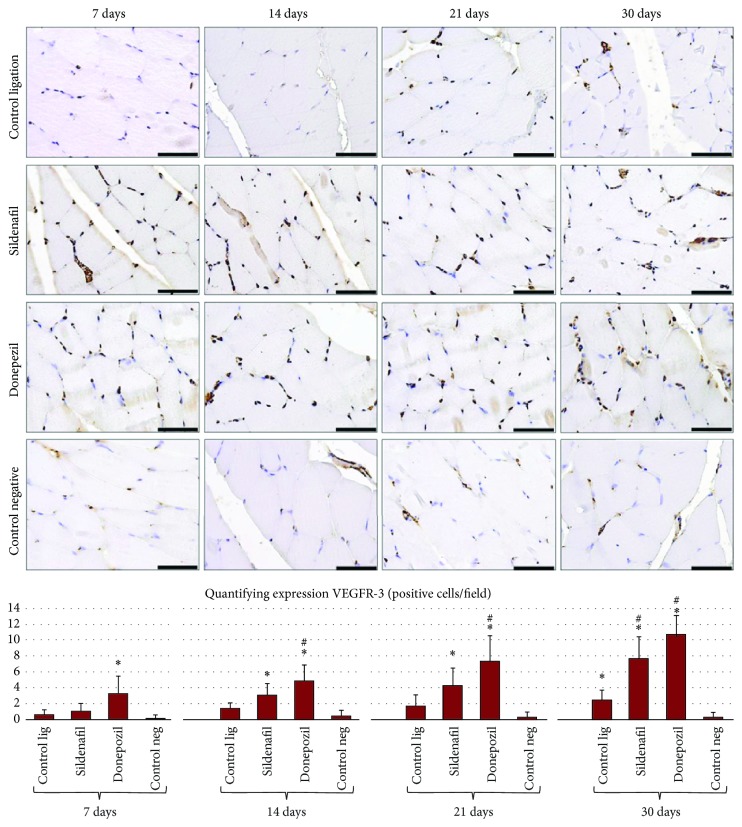
Histochemical evaluation of vascular endothelial growth factor in gastrocnemius muscle sections from Wistar rats, at the 4 experimental times (scale bar is 50 *μ*m). Quantification of the number of CD31 positive cells/field: the graph shows the mean and standard deviation for each experimental variant. Statistical differences are marked: ^*∗*^*P* < 0.0125 compared to the negative control group and ^#^*P* < 0.0125 compared to the ligation control group.

Following VEGFR-3 expression analysis, it was observed that, in the case of muscle sections from animals of the negative control group, very few positive cells were present (Figure 6). In contrast, in the case of animals of the ligation groups (regardless of treatment), an increased expression of vascular endothelial growth factor receptor 3 in small capillary structures was observed (Anova *P* < 0.001).

VEGFR-3 expression was significantly higher (*P* < 0.0125) following sildenafil treatment compared to the negative control group at 14, 21, and 30 days and compared to the ligation control group at 30 days. The same but more intense effect was found in the case of donepezil treatment, which caused an increase of VEGFR-3 expression that was statistically significant at all four experimental times compared to the negative control group and at 21 and 30 days compared to the ligation control group.

## 4. Discussions

Therapeutic angiogenesis and arteriogenesis remain an important goal in the case of patients with critical ischemia without revascularization possibilities. Many therapies have been attempted, with more or less encouraging effects [2, 17].

In this study, we demonstrated that sildenafil and donepezil treatment improves ischemic tissue perfusion by increasing angiogenesis and arteriogenesis.

Sildenafil is a strong inhibitor of phosphodiesterase 5 (PDE5), being used in the treatment of erectile dysfunction. PDE5 is found in smooth muscles of vessels and bronchi, in thrombocytes, cardiomyocytes, and skeletal muscle [19, 20]. It induces vasodilation by increasing cGMP concentration and changing NO availability and/or activity.

Previous studies have demonstrated that sildenafil treatment results in an increase of cGMP both in ischemic and nonischemic tissues, but increased blood flow has only been observed in ischemic tissues [21]. These results suggest the fact that sildenafil treatment can enhance the response of ischemic tissues to angiogenic and arteriogenic factors. It has also been suggested that sildenafil treatment increases the production of growth factors with an angiogenic effect [21].

Sildenafil is an inhibitor of PDE5, which catalyzes the breakdown of cGMP, one of the primary factors that cause smooth muscle relaxation. It has a potent action of enhancing NO driven cGMP accumulation. Other effects of cGMP on the vascular system are a decrease of cellular adhesion molecules, a decrease of platelet adhesion and aggregation, decreased activity of inflammation markers, and decreased leukocyte adhesion and diapedesis.

Furthermore, NO is supposed to be involved in angiogenesis through the PI3Akt-hypoxia-inducible factor- (HIF-) 1*α* pathway. VEGF plays a key role in the angiogenic process, and its expression is activated by ischemic tissues, under conditions of decreased oxygen levels, which results in enhanced expression of HIF-1*α* [13, 22]. NO is a potent vasodilator, playing a critical role in modulation of vascular tone. Kuwabara et al. proved the involvement of NO in this pathway using a NO donor, S-nitroso-N-acetylpenicillamine (SNAP), inducing HIF-1*α* and VEGF production in cardiomyocytes.

In our study, we demonstrated an increase in the number of nuclei in structures compatible with capillaries following sildenafil treatment, the increase becoming significant at 30 days (*P* < 0.0125). At all four experimental times, the number of nuclei/field in the gastrocnemius muscle was significantly reduced compared to animals of the negative control group. Rinaldi et al. also demonstrated that sildenafil potentiates the effect of hypoxia on angiogenesis in trained rats, emphasizing its proangiogenic effect [22].

Following donepezil treatment, the number of capillary structures was significantly lower compared to the negative control group at all four experimental times but was significantly higher compared to animals of the ligation control group (without treatment) at 7, 21, and 30 days (*P* < 0.0125).

No statistically significant differences were found between sildenafil and donepezil treatment, although at all four experimental times the values of the number of nuclei/field were higher after donepezil treatment.

The in vivo proangiogenic effect may occur by direct action or through proangiogenic factors [20, 23]. Neovascularization was evaluated by analysis of capillary density (CD31 positive cells) and the capillary/muscle fiber ratio. These were significantly increased in the case of rats treated with sildenafil at 7 and 21 days compared to the group of untreated animals, demonstrating the implication of sildenafil in the initiation of angiogenesis by formation of new capillaries, in a time dependent manner. This effect was observed in previous studies, by Senthilkumar et al., who demonstrated increased angiogenesis in animals treated with sildenafil [21]. Another study demonstrated the implication of sildenafil in angiogenesis, not only in the increase of capillary density, but even in the increase of arteriolar density [24].

It is suggested that cholinergic interventions may be involved in angiogenic and also in myogenic reactions [25]. Donepezil, as an acetylcholinesterase inhibitor, improves tissue perfusion and attenuates ischemia-induced muscle atrophy [16].

Donepezil treatment influenced to a greater extent the angiogenesis process, causing a significant increase (*P* < 0.0125) in the number of capillaries/mm^2^ compared to the ligation control group at 7, 21, and 30 days and compared to the sildenafil group at 21 days. Kakinuma et al. also studied the proangiogenic effect of donepezil; the authors suggested the fact that donepezil enhances angiogenesis in the ischemic limb, by modulating the acetylcholine level in the cells through a receptor dependent or independent mechanism, and acetylcholine derived from these cells might play a role in angiogenesis [16]. The same tendency was observed in the case of the capillary/muscle fiber ratio. Among the treatment groups, the capillary/muscle fiber ratio was the highest in the case of donepezil treatment. At 30 days, this treatment significantly influenced (*P* < 0.0125) the capillary/muscle fiber ratio, which was increased at this time point compared to untreated ligated animals (ligation control group).

It was also found that the capillary/muscle fiber ratio gradually increased with the time elapsed from ligation, in all 3 ligation groups.

Endothelial cells were assessed by evaluating the number of Ki-67 positive cells (dividing cells) and quantifying VEGFR-3. While these were very low in the gastrocnemius muscle from the nonligated limbs, in the ligated limbs, significant differences were found at all four experimental times (Anova *P* < 0.001).

Sildenafil treatment led to a progressive increase in the number of dividing cells (at 7, 14, 21, and 30 days). VEGFR-3 expression was significantly higher (*P* < 0.0125) following sildenafil treatment compared to the negative control group at 14, 21, and 30 days and compared to the ligation control group at 30 days.

VEGF was associated with the initiation of the angiogenesis process, by endothelial cell recruitment and proliferation. However, the mechanism by which sildenafil induces an increase of VEGF is not completely understood. Several hypotheses have been advanced: (1) sildenafil activates KATP channels and induces nitrate-like effects; it may increase tissue adenosine production through NO or the activation of ecto-5-nucleotidase mediated by KATP channels [26]. It is considered that adenosine enhances VEGF protein synthesis and mRNA expression through A2R. Endogenous adenosine may increase basal VEGF secretion in vascular smooth muscle cells, playing an important role in angiogenesis and arteriogenesis [27]; (2) sildenafil, which activates both eNOS phorphorylation and expression, may contribute to the regulation of VEGF expression. It was reported that eNOS deficiency results in a diminution of myocardial capillary growth and a reduction of VEGF expression in the myocardium of newborn mice [28].

The same effect of increasing VEGFR3 expression, but in a more intense manner, was observed in the case of donepezil treatment, which induced a statistically significant elevation of VEGFR-3 expression at all four experimental times compared to the negative control group, and at 14, 21, and 30 days compared to the ligation control group. Previous studies demonstrated the effect of donepezil in increasing VEGF expression and initiating angiogenesis. The effect of donepezil was inhibited by atropine, a muscarinic receptor antagonist [13]. The mechanisms by which donepezil accelerates angiogenesis probably involve the action of acetylcholine and nicotine, whose proangiogenic effect was reported in previous studies [29–31]. Both our results and those of Kakinuma et al. suggest that donepezil activates angiogenesis under ischemia conditions, by increasing the levels of angiogenic factors, enhancing proliferation, inhibiting apoptosis, and diminishing ischemia-induced muscle atrophy [16], through insufficiently known receptors. Thus, donepezil activates VEGF protein expression, accelerating endothelial cell proliferation, and inhibiting apoptosis, partially independently of cholinergic receptors. Moreover, donepezil has an effect on ischemic muscle atrophy by activating satellite cells to produce VEGF protein [32]. Other authors reported negative results of donepezil. Miyazaki et al. reported the antiangiogenic effect of donepezil, by inhibiting inflammation, after administration of higher doses [33]. We can explain the opposite effect by the inhibiting action on acetylcholinesterase after ad libitum administration in mice with experimentally induced critical ischemia, with attenuation of neovascularization, mRNA expression and proteins. In our study, the dose was strictly controlled by gavage administration.

For the sham group, there was a significant decrease in the capillary/muscle fiber ratio in the ligated versus the nonligated limb. The same trend was seen for both sildenafil and donepezil treatments, but to a lesser degree. This is similar to previous reports showing that, after 8 weeks of administration, in normal nonischemic muscle, sildenafil can result in both promotion of angiogenesis (increased capillary density) and reduced muscle fiber size [22]. In the gastrocnemius muscle sections from the nonligated limbs, the number of dividing (Ki-67 positive) cells was very low, unlike in the muscle sections from the ligated limbs; sildenafil augmented this effect, and donepezil even more obviously so, but not at the level of the ischemic limb. Following VEGFR-3 expression analysis, it was observed that, in the case of muscle sections from animals of the negative control group, very few positive cells were present, contrasting with animals of the ligation groups (regardless of treatment), in wich case an increased expression of vascular endothelial growth factor receptor 3 in small capillary structures was observed.

The improvement in angiogenesis, found in our study, after sildenafil and donepezil administration, can be based on NO biosynthesis through an indirect cholinergic mechanism. Sildenafil administration, even if it has no effect on Ach biosynthesis, can result in an increase of Ach and NO production through noncholinergic mechanisms [33]. The superior angiogenic effects of donepezil can be attributed to cholinergic mechanisms, both Ach inhibiting and non-Ach.

## 5. Conclusions

Capillary density and angiogenesis assessed based on the number of CD31 positive cells, Ki-67, and VEGFR-3 immunoreactivity were significantly increased in endothelial cells from gastrocnemius muscle sections by sildenafil and donepezil treatment compared to controls, which indicates the fact that donepezil, to a greater extent than sildenafil, activates angiogenesis by increasing the levels of angiogenic signals in endothelial cells. Thus, it seems that donepezil treatment has a better effect in ligation-induced ischemia compared to sildenafil, promoting angiogenesis in the first place, and also arteriogenesis.
